# Nestedness-Based Measurement of Evolutionarily Stable Equilibrium of Global Production System

**DOI:** 10.3390/e23081077

**Published:** 2021-08-19

**Authors:** Jiaqi Ren, Lizhi Xing, Yu Han, Xianlei Dong

**Affiliations:** 1 College of Economics & Management, Beijing University of Technology, Beijing 100124, China; renjq@emails.bjut.edu.cn (J.R.); hanyu@bjut.edu.cn (Y.H.); 2 International Business School, Beijing Foreign Studies University, Beijing 100089, China; 3 Business School, Shandong Normal University, Jinan 250358, China; sddongxl@sdnu.edu.cn

**Keywords:** global value chain, global economic integration, nestedness, evolutionarily stable equilibrium

## Abstract

A nested structure is a structural feature that is conducive to system stability formed by the coevolution of biological species in mutualistic ecosystems The coopetition relationship and value flow between industrial sectors in the global value chain are similar to the mutualistic ecosystem in nature. That is, the global economic system is always changing to form one dynamic equilibrium after another. In this paper, a nestedness-based analytical framework is used to define the generalist and specialist sectors for the purpose of analyzing the changes in the global supply pattern. We study why the global economic system can reach a stable equilibrium, what the role of different sectors play in the steady status, and how to enhance the stability of the global economic system. In detail, the domestic trade network, export trade network and import trade network of each country are extracted. Then, an econometric model is designed to analyze how the microstructure of the production system affects a country’s macroeconomic performance.

## 1. Introduction

Being relatively independent for a long period of time, the production systems of countries and regions in the world have gradually formed a global economic system through flourishing trade. Numerous industrial value chains (IVCs) scattered among countries or regions constitute the global value chain (GVC) network, which is an interconnected and organic whole with specific functions. In such a network, the industrial sectors of each country form an interdependent and competitive community of shared destiny through the flow of capital, material and information.

**Figure 1:**
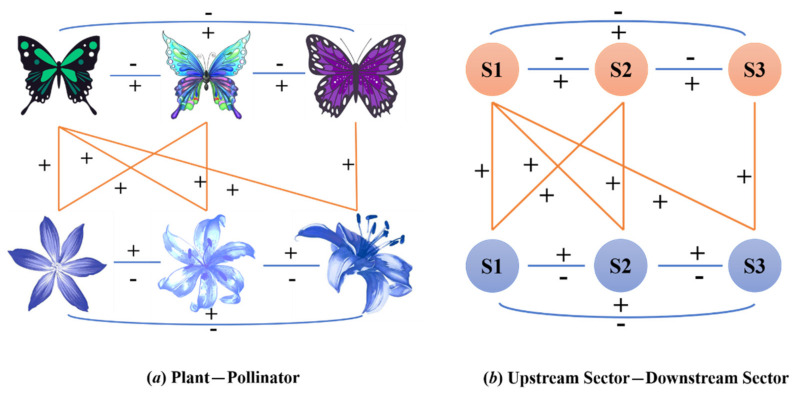
Comparison between Mutualistic System and Global Production System. (**a**) There exists a mutually beneficial symbiosis relationship between plants and pollinators. In simple terms, pollinators pollinate plants to promote the formation of their fruits and take in the nutrients they need at the same time. Among pollinators, there is not only competition for plants but also collaboration to complete the process of collecting pollination, which will be beneficial to both sides as their population grows. (**b**) Refers to the global production system where the orange circles represent the upstream sectors and the blue circles the downstream sectors. The numerous upstream and downstream sectors on the GVC cooperate to complete the industrial division of labor, while the upstream sectors not only compete for the same buyers but also collaborate to make sure these buyers can get what they need in the production process.

In theoretical ecology and evolutionary biology, “nestedness” refers to a structural measure of the overall stability in the ecosystems [1,2,3]. The structure is an optimal system state conducive to both sides, which is the result of a mutual benefit mechanism established between species and between species and the environment through evolutionary games [4,5]. Mutualistic species strengthen cooperation through network reciprocity and weaken competition by reducing niche overlap, so as to promote the system to an evolutionary equilibrium. This concept has, in recent years, also begun to be applied by sociologists and economists to analyze various phenomena related to human society. In the same way as the ecosystem, the economic system seeks an evolutionary equilibrium in the process of dynamic evolution. In today’s highly developed globalization, cooperation among economies has reached an unprecedented level. Through dynamic games to allocating scarce resources, economies in the global value chain can maximize their relative interests. The role of network reciprocity in emerging cooperation is an important mechanism for the global economic system to achieve dynamic balance [6]. As the leading link in the global value chain, the flow of intermediate products depends on the cooperation between various industrial sectors. Considering that the industrial sector in the GVC has a dual identity, the provider and consumer of intermediate products, it can be represented by a bipartite graph to separate the two attributes of a node, and, thus, the mutualistic relationship between upstream and downstream industries can be clearly depicted. In fact, the ecosystem and the global production system have some common ground. For instance, both the flow of energy between species and the flow of intermediate goods between industrial sectors reflect the mutually beneficial symbiosis relationship produced through the competition and cooperation game, as shown in Figure 1. With the nested structure being identified among industrial sectors in the GVC network, nestedness can be applied to measure the topological stability of both the whole and local parts of the global industrial ecosystem.

The ecological metaphor is not ecological reductionism or ecological imperialism, and it is not to simply reduce the phenomenon of macroeconomic evolution (industrial transfer between countries and adjustment of industrial structure within countries) to ecological evolution [7]. Moreover, Chase and Leibold’s research on the ecological niche is only an abstract milieu interne adjustment mechanism, and does not describe the specific evolution process. The complex system theory must be embedded in it to accurately explain the law of the evolution of an economic system [8]. Therefore, the evolutionary game theory of biological populations in ecology has a certain enlightening significance to the theory of economic evolution.

## 2. Literature Review

Nestedness, derived from theoretical ecology and evolutionary biology, is an important structural feature of complex networks. In 1957, Darlington mentioned this concept in his book *Zoogeography*, in which he noticed that the spatial distribution of species displayed the nested feature [9]. Building on Darlington’s discovery, in 1986, Patterson and Atmar formulated the precise conce pt of nestedness, i.e., in a fully nested network, the neighborhood of a node with lower node degree is a subset of the neighborhood of a node with higher node degree [10]. In 2003, Bascompte et al. analyzed 25 plant–pollinator networks and 27 plant–frugivore networks and found that most of the networks exhibited nested features [11]. The nested structure reflects the mutualistic relationship between species. In a mutualistic network, specialist species tend to relate to generalist species who have higher adaptability to the environment, thus mitigating the risk of extinction. Niche overlap decreasing in the nested structure helps weaken competition and improve species diversity [12], and the greater the nestedness, the stronger the recovery ability of the system after external shocks and the stabler the network structure [13,14,15].

Inspired by this discovery in ecological networks, scholars in socioeconomic networks began to devote themselves to the study of nestedness. As early as 1965, when studying the U.S. economic structure using intra-country input–output data, Leontief identified the obvious nestedness of the U.S. industrial network [16]. Subsequently, a large number of theoretical and empirical studies have emerged, which has greatly enriched the interdisciplinary research in the field of economics. The world trade network [17], the arms trading network [18], the interbank capital flow network [19], the manufacturer–supplier network [20] and the product export network [21,22,23] also show nested features. The network heterogeneity caused by the dynamic evolution of a social economic system is the main reason for its nested structure. Taking the world trade network as an example, the coexistence of competition and cooperation among countries leads to unbalanced economic development, which makes the world trade network show a center–periphery structure. That is, the generalist sectors are connected with the most counterparts and form the core of the network, and specialist sectors are at the periphery and are dependent on the center [24,25]. This highly connected center makes the links of the network replaceable. Even if the supply or demand of some sectors disappears, the existence of other replaceable sectors can make the products flow normally. At the same time, these studies reflect that the nested structure is of great significance for maintaining the stability of the economic system [26,27]. For example, in the 2008 global financial crisis, the reason for the decrease in interbank transactions was that the core banks reduced the number of externally active sides [28].

This paper is organized as follows. In Section 1, the application and development of nested structure theory in the field of ecology and economics are systematically introduced. In Section 2, we sort out the literature on nested structure in economic system and ecosystem. In Section 3, a GVC network is built based on the Multi-Region Input–Output (MRIO) database to embody the flow of intermediate goods between industrial sectors. In Section 4, the nested structure is embodied by sorting algorithm and measured by NODF method. In Section 5, analyses of the divergence, trend and stability are conducted to explain the complex relations between industrial sectors and the global production system, and then the economically evolutionary mechanism is proposed. In Section 6, the econometric models are used to analyze the relations between the nestedness-based indicators and the level of economic development. Finally, some countermeasures are put forward for economies to achieve a much more stable and healthy state.

## 3. Data and Model

In order to represent the nested structure of the global production system, we build a GVC network that can reflect the coopetition relationships of industrial sectors in each country.

### 3.1. Data Sources and Structure

Among the mainstream databases, the Eora Multi-Region Input–Output (MRIO) database of the University of Sydney covers the largest number of economies (189 countries/regions) and the longest period (1990–2015) and is suitable to be taken as modeling data. To make the nested structure more explicit and hierarchical, a simplified version of this database, named Eora26, is chosen in this paper, i.e., to sort the industrial sectors in 189 countries/regions into 26 sectors. For the sake of effectively visualizing the data element, we further group these sectors into four sectoral categories, namely agriculture (sectors 1 and 2), mining (sector 3), manufacturing (sectors 4 to 14), and services (sectors 15 and 16) (For detailed dividing criteria, refer to Appendix A).

### 3.2. Network Modeling

As an emerging yet important research area, GVC accounting is mainly represented by teams of Timmer M.P., Koopman R. and Wang Z., who have made important breakthroughs in economic theories and statistical techniques [29,30,31]. The important quantitative results they obtained have enriched the original GVC studies and cemented a theoretical basis for both the upcoming analysis and the formulation of relevant policies. They have also enabled the theoretical expansion to other GVC-related fields. Among all the achievements in GVC accounting, a set of preliminary accounting systems has been formed around value-added exports, which reflects industrial sectors’ competitiveness and participation in the GVC.

The global economic system, however, is a complex nonlinear emergence system, and the multiple emergences as its essential feature cannot simply be obtained by the linear addition of individualities. That is to say, the whole picture will be shadowed if only the individuals are analyzed. We should focus on the interrelationship and influence mechanism between individuals and the whole, under the perspective of systems science.

**Figure 2:**
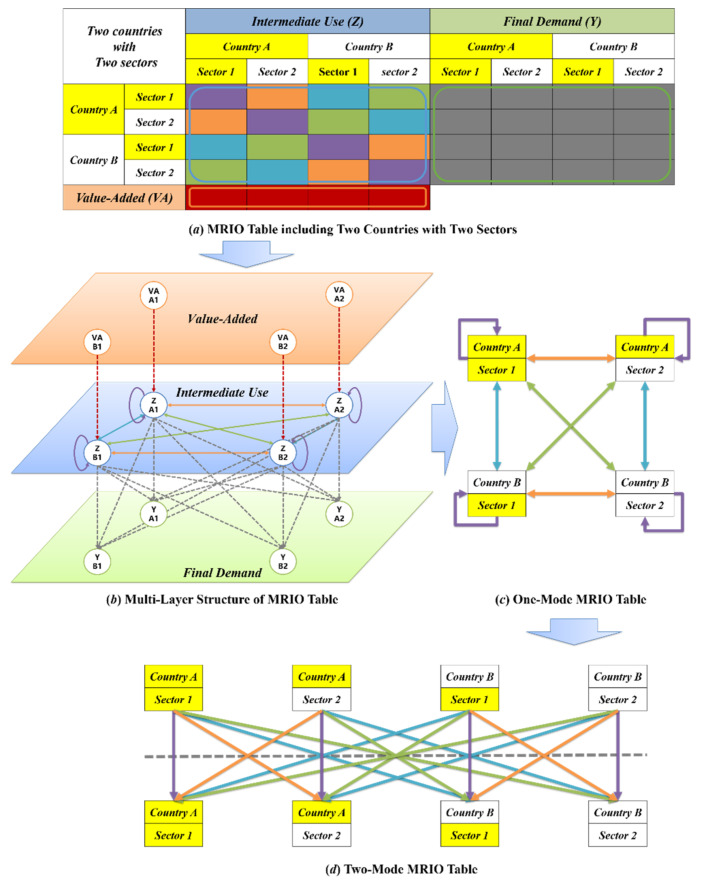
Relationship between MRIO Table and Network. Typical MRIO table includes three different areas, namely value-added, intermediate use and final demand. It is possible that the whole global economic system can be abstracted to a multilayer network as shown in Figure 2b, which includes three layers: the value-added layer, the intermediate use layer, and the final demand layer. The intermediate use layer can be further treated as a puzzle that is made of many single-layer networks out of a multilayer network, in which the nodes are the countries/regions, the layers are the industrial sectors, and links can be established from sellers to buyers within and across industrial sectors. If necessary, we can change the one-mode MRIO network into a two-mode network to separate the inner identity of each sector and prepare for the projection. In Figure 2d, the same sector distributes on the two sides of the dotted line, which means it belongs to both the upper stream and the lower stream. In other words, the upper stream sector in the MRIO table could be referred to as the object nodes in the bipartite graph, while the lower stream sector could be the participant nodes. Now, the self-loop is transformed into an edge between the two identities of this sector.

The fact that industrial sectors in the GVC function as both upstream and downstream sectors can be displayed in a bipartite graph, as in Figure 2d. Based on the data of intermediate use in the MRIO table, a Global Industrial Value Chain (Bipartite Graph) Network (GIVCNBG) is constructed in the form of a bipartite graph $G=\left(O,P,E,W\right)$. In the *G*, all upstream industrial sectors form the set of object nodes O, and all downstream industrial sectors form the set of participant nodes P; edges pointing from upstream to downstream form the set of edges E in competition with other N-1 sectors as a consumption sector, downstream industry sector *i* obtains from its upstream industry sector j intermediate goods input, the amount of which is$\;w_{ji}$ (j= *i* indicates that the upstream and downstream are the same sector), constituting the weight set W.

While the GIVCNBG model applies the weight set W to substitute the adjacency matrix, each row refers to the distribution of intermediate goods output of an upstream sector and each column the intermediate goods input of a downstream sector. In spite of this being the same mechanism as the one-mode GIVCN model, a two-mode network is able to identify the underlying cooperative relationships between industrial sectors. That is, there is cooperation between upstream sectors to promote the production of their common downstream sectors [32]. The authors believe that the flow of intermediate goods in production systems (expressed in the IO table as value or currency flow) is similar to the flow of energy in ecosystems, and that both systems converge to a steady state after a complex game. As mentioned above, ecological studies have found that ecosystems in a steady state are characterized by nested structures and a more stable mutualistic relationship between species. Therefore, we believe such features can also be found in the topology of production systems due to them having the same evolutionary mechanism.

### 3.3. Network Pruning

The GIVCNBG model is an extremely dense weighted network with highly heterogeneous material flows between each upstream sector and downstream sector. Thus, it needs to be pruned in search of the backbone part before nested structure analysis. In this paper, a new heuristic algorithm—XIFA [33,34]—is proposed, with the integration of the features of H-index [35], Pareto Principle and Disparity Filter [36]. Through XIFA, a special subgraph $\overset{´}{G}=\left(O,P,\overset{´}{E},\overset{´}{W}\right)$ is extracted from the GIVCNBG model, named as the GIVCNBG-FE model (FE as in “Filtering Edges”).

The GIVCNBG-FE model compresses the size of the edge set E to a large extent, For example, after pruning the GIVCNBG-Eora26SC4-2015 model by XIFA, $\left|\overset{´}{E}\right|=8.95\%\times \left|E\right|$, while $\sum \overset{´}{w_{ij}}=99.15\%\times \sum w_{ij}$, which means more than 90% of the deleted edges carry less than 1% of the network information, leaving less than 10% of the edges carrying more than 99%. In sum, the bipartite graph $\overset{´}{G}=\left(O,P,\overset{´}{E}\right)$, with the weight information removed and all edges left as important, is sufficient to portray the nested structure of the network (For detailed algorithm information, refer to Appendix B).

## 4. Measurement

A nested structure is determined by the distribution of edges in the network and can be influenced by the network connectivity. The higher the connectivity of the network, the more likely it is to exhibit nested characteristics. In ecosystems, nested structure is established when ecological niches of different species adapt to each other and, thus, achieve dynamic equilibrium. It is a network structure characteristic formed by species adapting to the natural environment in pursuit of homeostasis. Nestedness in an ecological network is therefore a measure of the stability and sustainability of an ecological environment. From the perspective of bionomics, there are many similarities between a GVC network and an ecological network in terms of topological characteristics. In the same way as biological species, the industrial sectors in the GVC form a complex association of mutual benefit, and the trade and economic cycles between them make the GVC an organic whole. Higher nestedness of the GVC network indicates a more mature industrial trade mechanism, a more regular and orderly industrial trade network and a deeper integration between industries. Hence, research on the nested structure of the GVC network has fundamental implications for the economic development of countries, regions and even the world [37].

Prior to the analysis, the adjacency matrix needed to be reordered to maximize the degree of network nestedness. Several classical sorting algorithms are introduced in Appendix C, and the SBD (sorted by degree) algorithm was finally selected through comparing their NODF. The NODF metric proposed by Almeida-Neto et al. is widely used to calculate the nestedness of networks, which is based on two basic properties: Decreasing Fill (DF) and Paired Overlap (PO) [38].

Given that a matrix has m rows and n columns, and MT is the number of elements valued at 1 in any row or column. For any pair of rows $\left(i,\;j\right)(i<j)$, if $MT_{i}>MT_{j}$, then $DF_{ij}=100$, otherwise $DF_{ij}=0$; similarly, for any pair of columns $\left(k,\;l\right)(k<l)$, if $MT_{k}>MT_{l}$, then $DF_{kl}=100$, otherwise $DF_{kl}=0$.

For any pair of rows $\left(i,\;j\right)(i<j)$, $PO_{ij}$ refers to the percentage of 1′s in a given row j that is located at identical column positions to the 1′s observed in a row i. Similarly, for any pair of columns $\left(k,\;l\right)(k<l)$, $PO_{kl}$ refers to the percentage of 1′s in a given column l that is located at identical row positions to those in column k. Therefore, for any up-to-down row pair, or any left-to-right column pair, the degree of paired nestedness ($N_{paired}$) can be expressed as follows

(1) $$N_{paired}=\left\{\begin{array}{c} 0,\;if\;DF_{paired}=0\\ PO,\;if\;DF_{paired}=100 \end{array}\right.$$

There are $m\left(m-1\right)/2$ row pairs in row m, and $n\left(n-1\right)/2$ column pairs in column n. Thus, the nestedness of the entire network can be calculated by “averaging all paired values of rows and columns”

(2) $$NODF=\frac{\sum N_{paired}}{\left[\frac{m\left(m-1\right)}{2}\right]+\left[\frac{n\left(n-1\right)}{2}\right]}$$

where the *NODF* value ranges from 0 to 100, with NODF=0 indicating a non-nested network structure and NODF=100 indicating a fully nested network structure.

## 5. Results

Globalization is both an opportunity and a threat for the economic development of each country. On the one hand, the industrial sectors of each country have their comparative advantages, thus forming a relatively stable international industrial division of labor. On the other hand, they also fiercely compete in the global market, seeking a place on the GVC. It is under the impetus of both cooperation and competition that the global economic system evolves and shows nested structural characteristics in the process of convergence to homeostasis.

### 5.1. Divergence Analysis

If the economic system is compared to an ecosystem, the generalist feature of an industrial sector can be measured by the number of important IO relationships they establish with other sectors. In this paper, the larger-degree industrial sectors are defined as Generalist Sector, featuring higher involvement in the GVC, widely distributed outputs/inputs and a broader industrial ecological niche. On the opposite side are the Specialist Sector. Viewed by rows, the nodes in the upper part of the nested area have higher outdegree and stronger supply-side generalist degree, while those in the lower part have lower outdegree and weaker supply-side generalist degree. Viewed by columns, the nodes on the left side have higher indegree and stronger demand-side generalist degree, while those on the right side have lower indegree and weaker demand-side generalist degree.

Due to the vertical specialization, product manufacturing, and its related services, exists through all stages of the global production process. Each country takes advantage of its own and others’ comparative advantages in technology, capital and/or labor, jointly shaping the main structure of the GVC. As a result, the manufacturing and services sectors own a higher degree of external dependence, i.e., a higher generalist degree. In contrast, the agriculture and mining sectors only affect a limited number of sectors. They mainly trade with the domestic sectors for self-sufficiency and seldom establish international trade channels with the manufacturing sectors of a few developed economies. In short, most of them have low involvement in the GVC, resulting in lower generalist degree.

**Figure 3:**
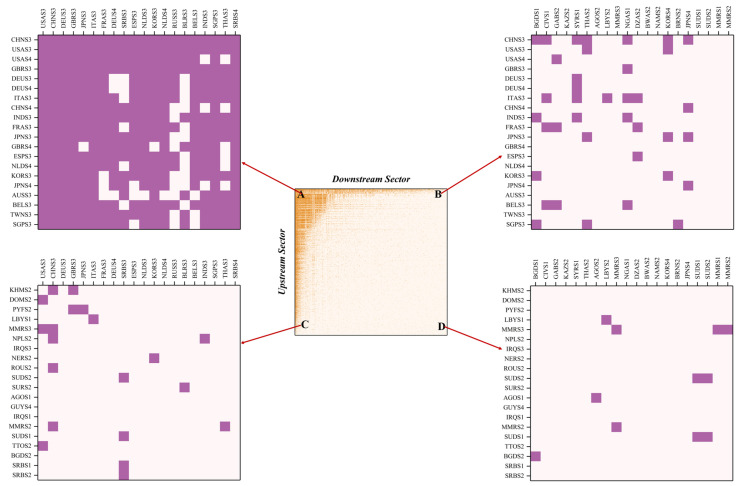
Topology of Different Regions after Sorting the Adjacency Matrix of GVC Network Based on SBD Algorithm. (**A**) The IO relationship between the upstream and downstream generalist sectors; (**B**) The IO relationship between the upstream generalist sectors and the downstream specialist sectors; (**C**) The IO relationship between the upstream specialist sectors and the downstream generalist sectors; (**D**) The IO relationship between the upstream and downstream specialist sectors.

To further analyze the features of the nested structure of the GVC, this paper selected four representative areas consisting of the top twenty and bottom twenty sectors on the supply and demand sides, respectively, as shown in Figure 3. The comparison reveals significant differences in the generalist degree of industrial sectors in developed and developing countries.

Region A on the top left shows the IO relationship between the upstream and downstream generalist sectors. This value network, consisting of manufacturing and services sectors in advanced economies, is very dense, indicating that intense competition occurs because of overlapping ecological niches. The NODF value of Region A is 76.165, due to the empty elements of the upper triangle and the non-empty elements of the lower triangle, which indicate the insufficient collaboration and excessive competition among these generalist sectors; hence, negatively affecting the stability of the industrial structure.

Region B in the top right shows the IO relationship between the upstream generalist sectors and the downstream specialist sectors. It is found that Japan’s services belong to the generalist sector in the upstream, whose trade in services exports to most countries around the world, while they have very low generalist degree in the downstream. On the one hand, Japan’s services sectors are highly developed and are mainly in the form of outsourcing. Along with the progress of economic globalization and division of labor, they penetrate every aspect of the global market. On the other hand, Japan’s market remains relatively closed. Since World War II, the industrial structure of Japan has been continuously upgraded, and various industries, especially the services sectors, have entered into a relative mature stage and become the dominant industry. As for other countries, it is difficult to compete with Japanese companies because of the high trade barriers.

From the bottom two regions, the agriculture and mining sectors in underdeveloped countries/regions have lower generalist degree; except for achieving self-sufficiency (Region D), they only open international trade channels with the manufacturing sectors of a few developed economies (Region C). On the one hand, as the multilateral trading system is frequently challenged by unilateralism, agriculture sectors often passively become an important bargaining chip for balancing bilateral economic and trade relations, together with the presence of invisible barriers to agricultural trade, posing obstacles to the globalization process of the agricultural sector. On the other hand, since the globalization of the mining sectors depend on resource endowment and geographical factors, only a few countries are able to achieve significant exports of mineral resources, upon which most other countries have to rely.

### 5.2. Trend Analysis

**Figure 4:**
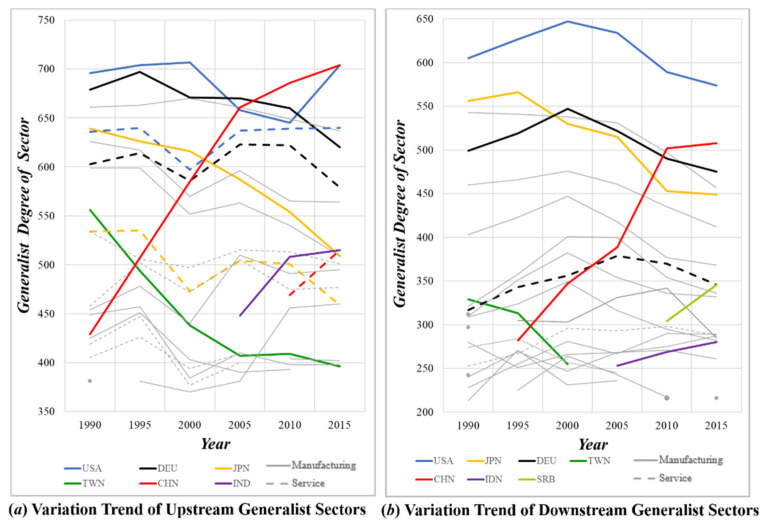
Generalist Degree Variation Trends of the Upstream and Downstream Industrial Sectors.

To observe the dynamic trend, this paper put together the top twenty industrial sectors in terms of generalist degree on both sides, as shown in Figure 4. Overall, the major generalist sectors did not change significantly between 1990 and 2015, with the absolute generalist value fluctuating on a small scale. In particular, the manufacturing and services sectors of the U.S. and Germany in the export sectors, and their manufacturing sectors in the import sectors, have always maintained a high generalist degree, which means these sectors are deeply integrated into all parts of the global economic cycle. In addition, some variation trends also deserve more attention.

First, on the matter of the export trade reflected by the upstream sector’s generalist degree or the import trade reflected by the downstream sector’s generalist degree, these tend to wax and wane. It is clear that, with the scaling-up influence exerted by China’s manufacturing export trade, the generalist degree distribution of the upstream manufacturing sector has evolved from a “U.S.–Germany–Japan” tripolar pattern to a “China–U.S.–Germany” pattern [39]. Meanwhile, Chinese exports of trade in services have begun to narrow the gap with developed countries and have surpassed Japan.

Second, the rise of manufacturing sectors on the Chinese mainland and India have brought an impact on Taiwan. As one of the once “Four Asian Dragons”, Taiwan used to be a supply chain hub in Asia, except for Germany and Japan, for western countries. However, with the advent of the dividends of Chinese reform and their opening up, productive enterprises in Taiwan began to move to the Chinese mainland and overseas, leading to the significant decrease in influence of Taiwan’s manufacturing industry. Besides, in order to accelerate the development of the manufacturing industry, the Indian government has introduced a batch of relevant measures to stimulate investment and ease market access for foreign investment. Due to the blockade and restrictions imposed by the European and American markets on the Chinese market, a huge market such as India is taken as the preferred place for partial industrial transfer, which provides favorable conditions for the development of manufacturing industry in India [40].

Third, Serbia has become a new “European Factory” by virtue of its unique location and has started to play an important role in the import and export trade of manufacturing industry in recent years. Located at the junction of the East and the West, Serbia is an important hub connecting the major corridors of Europe and Asia and boasts strong connectivity. Besides, it has signed free trade agreements with the European Union and Central and Eastern Europe, and enjoys the most-favored-nation treatment from the U.S. With the progress of the Belt and Road Initiative, Chinese enterprise also brings infrastructure construction, creating a favorable environment for the development of the Serbian manufacturing industry. All of these positive factors make Serbia an important intermediate goods processing link in the GVC.

### 5.3. Stability Analysis

**Figure 5:**
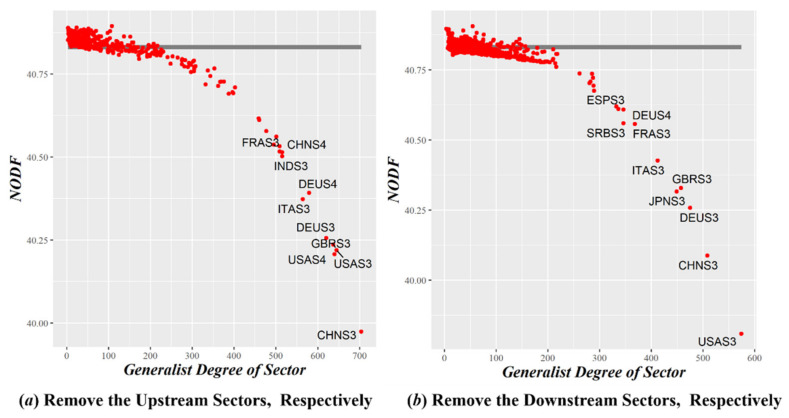
The NODF of Removing a Certain Industrial Sector of GVC Network. The horizontal gray lines represent the NODF of the nested network sorted by the SBD algorithm, and the red scatter points represent the correspondence between the generalist degree after removing a certain industrial sector (the size of the outdegree or indegree) and the new NODF of the nested network.

**Figure 6:**
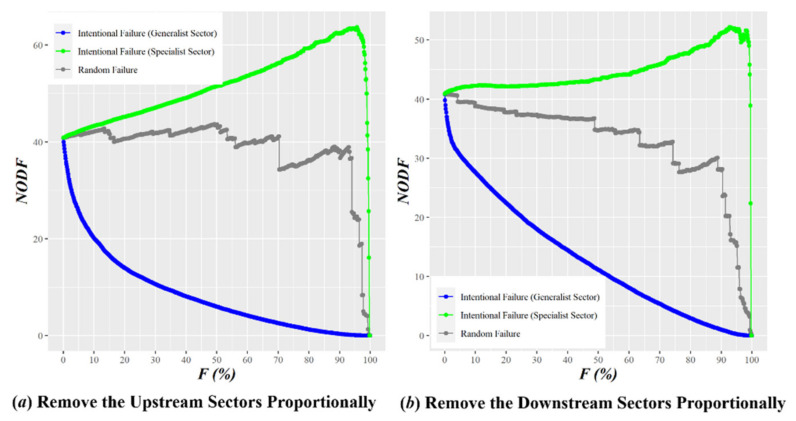
The NODF of Proportionally Removing Industry Sectors of GVC Network. The gray lines represent the variation in the value of network nestedness after randomly removing a certain proportion of industrial sectors from the aligned adjacency matrix; the blue lines represent the variation in the value of network nestedness after removing industrial sectors from the aligned adjacency matrix in the descending order of generalist degree; the green lines represent the variation in the value of network nestedness after removing industrial sectors from the aligned adjacency matrix in the descending order of specialist.

It is necessary to quantify the influence of generalist and specialist sectors on the nestedness of the GVC network, two control tests were designed to examine the influence of a certain sector and the cumulative influence of multiple sectors, respectively. The results are shown in Figure 5 and Figure 6.

After removing a generalist sector (see Figure 5), the NODF of the nested network significantly decreases, indicating that the higher the industry sector’s generalist degree, the more positive its effect on maintaining the stability of the GVC network. In contrast, after removing a specialist sector, the NODF slightly increases, which means that an industrial sector with a lower degree of generalist would weaken it. With the deepening of globalization, these industrial sectors risk being marginalized or even eliminated if they do not actively participate in international competition and cooperation.

Figure 6 further confirms the above findings. After removing a small proportion of industrial sectors with the highest generalist degree, the NODF of the nested network falls drastically, i.e., the stability of the GVC network deteriorates rapidly, indicating that a few generalist sectors are important hubs to maintain the functioning of the GVC. In contrast, when the industrial sectors with the lowest generalist degree are removed, NODF displays an increasing trend that does not start to decline until only 10% of the industry sectors are left. This reinforces the importance of the generalist sectors to stability.

By comparison of Figure 6a,b, we further find that there is a difference between generalist and specialist sectors in their terms of impact. Given the same proportion of removed generalist sectors (e.g., 10%), the removal of upstream sectors would exert a greater negative impact on the nestedness of the GVC network than that of downstream sectors. In other words, the global demand network of intermediate goods (consisting of downstream sectors) is sturdier than the supply network (consisting of upstream sectors). Considering this, when the global economy faces systematic risks, in order to cope with the resulting pressure or even disruptions of supply chains and reduced economic dependence on external resources, many countries have explored alternatives for supply chain management and import dependency. For example, they usually move their supply chains to countries less affected by the pandemic, pull some of the production capacity back from overseas or accelerate the industrialization process.

### 5.4. Evolutionary Mechanism

Dependency Theory, also known as Core-Periphery Theory, is established on the world trade pattern and the resulting unequal international division of labor. It is powerful as it explains the differences between developed and developing economies. The simple explanation is that developed countries gather at the center of the world economic system, while developing countries scatter at the periphery. Functionally, the countries at the center transfer the production of primary products to peripheral countries through capital import and transnational corporations, exploiting the peripheral countries’ cheap labor resources to develop labor-intensive industries and, thus, optimizing their own industrial structure. Being subject to the external constraints of the central countries, peripheral countries form dependence on the central countries and the surplus value keeps flowing from the periphery to the center, thus leading to the rich countries getting richer and the poor countries getting poorer [41,42,43,44]. For example, after World War II, the Asian, African and, especially, Latin American countries did not embark on the road to affluence after their attainment of independence but, instead, became even more dependent on, and formed the neo-colonialist industrial affiliation with, the economies of capitalist countries in Europe and North America. Given that, advocates of the dependency theory call for trade protection and import substitution in the peripheral countries and, with a strong nationalist tendency, encourage them to develop their own industries. However, this theory puts the peripheral countries in a passive position and attributes their economic distress on external factors, taking no consideration of the drawbacks in their domestic economic structures, which is to some extent pessimistic and biased.

**Figure 7:**
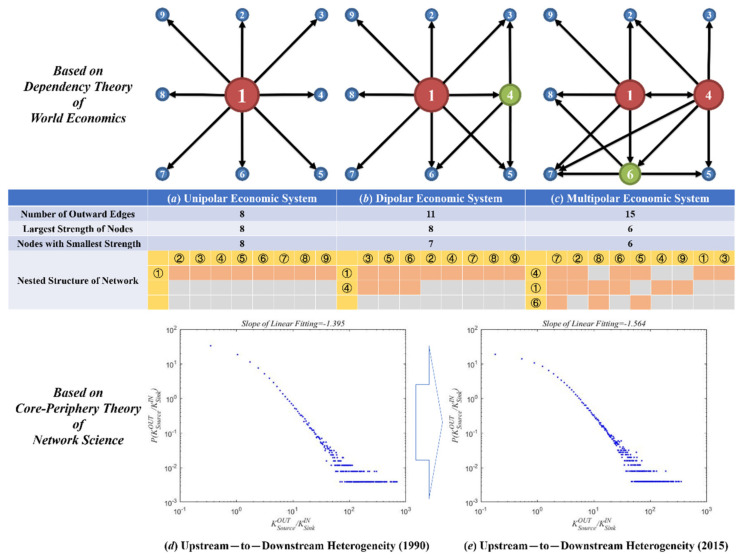
The Formation Process of Nested Structure of GVC Network. The orange circle 1 in (**a**) represents the developed countries initially at the center of the world economic system, while the yellow circles are the developing countries at the periphery, and the size of the circles reflects the degree of centrality of the countries; (**b**,**c**) indicate the gradual migration process of peripheral country 4 and peripheral country 6 to the central position, respectively. Besides, the ratio of outdegree of upstream sectors to indegree of downstream ones is designed to reflect the heterogeneity of development level of economies. Accordingly, the absolute value of slope of linear fitting increasing in (**d**,**e**) means our world is flattened by the economic integration.

Despite the similar nested structures of the mutual benefit ecosystem and the global economic system at the topological level, their formation mechanisms are not identical. Species in the mutual benefit ecosystem enhance their ecological benefits by continuously adjusting their interactions with other species, and the nested structure evolves through species’ dynamic game play and active adaptation to the environment. On the other hand, the driving force behind the formation of the core-periphery model of the global economic system lies in the international division of labor based on the countries’ comparative advantages and, therefore, features historical inevitability. However, from the perspective of dynamic development, peripheral countries are not always stuck in a position of being exploited and unable to develop their economies. On the contrary, the industrial transfer of the center countries creates opportunities for the peripheral countries to make full use of the capital and technology of developed countries to promote their own industrial development and technological innovation, thereby achieving so-called “Corner Overtaking”. With the increased depth and breadth of the GVC, by transferring many industrial production processes to developing countries, developed countries have completed the transition from a production-based society to a consumption-based society and need to rely on the supply of goods from developing countries. This has inevitably led to the emergence of industrial hollowing-out in some developed countries, increasing their dependency on developing countries as the world factories. Figure 7 briefly shows the movement of peripheral countries to the center of the industrial landscape, which is also the formation process of the nested structure of the global production network. From the perspective of evolutionarily economics, the continuously flattened world is derived by the evolutionarily stable equilibrium of the global production system.

Of course, the heterogeneity of economic development is still prevalent, and even increasingly serious. Even if the peripheral countries in the global industrial pattern achieve their economic growth targets, they are still at the end of high-tech diffusion, lacking core technologies and high value-added products, and are often subject to economic sanctions and technological blockade by central countries. In other words, the dependence of peripheral countries on the center countries is much stronger than that in the reverse. Developing countries therefore need to face up to the gap with developed countries in various aspects, transforming their economic growth mode so as to become the beneficiaries of economic globalization rather than just the contributors.

## 6. Econometric Analysis

Based on the above analysis, it is found that the generalist degree of a country’s industrial sectors is closely related to its economic status and productive capacity. Therefore, the focus of the following section is on whether a country’s economic condition is affected by the nested structure of the GVC network or, in other words, how a country’s macroeconomic performance is related to the microstructure of trade networks.

### 6.1. Correlation between Variables

**Figure 8:**
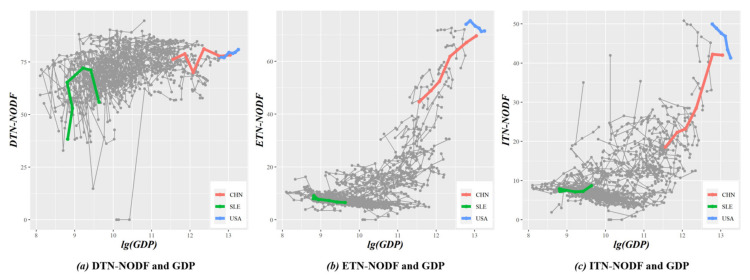
Correlation of DTN-NODF, ETN-NODF, ITN-NODF and GDP. Three countries with large differences in GDP are selected, with red representing China, blue the United States and green Sierra Leone. Data source: World Bank—https://data.worldbank.org.cn/indicator, accessed on 14 January 2021.

Considering that a country’s macroeconomic performance is affected by both domestic and international trade cycles, this paper designs three NODF-based indicators to measure the nestedness of the local network in terms of economies. Firstly, DTN-NODF measures the nestedness of the home trade network, which consists of trade activities of intermediate products between industrial sectors within a country. Secondly, ETN-NODF measures the nestedness of the export trade network, which is formed when industrial sectors of a country, as upstream sectors (supply side), trades intermediate products with other countries. Thirdly, ITN-NODF measures the nestedness of the import trade network formed when a country’s industry, as a downstream sector (demand side), trades intermediate goods with other countries. In terms of macroeconomic performance, this paper uses Gross Domestic Product (GDP) data provided by the World Bank. The correlation diagrams of these are plotted at intervals of five years, as shown in Figure 8.

First, in all countries, the values of DTN-NODF are larger (mostly between 50 and 80), and those of ETN-NODF and ITN-NODF are smaller (mostly between 0 and 20). This is because, compared with international trade networks, most countries have relatively mature domestic trade networks, in which domestic industrial sectors can also form synergy. Hence, it is much less difficult and risky to form a domestic trade cycle of industrial chains, moving the original country-to-country trade to a province-to-province and city-to-city economic cycle. Certainly, the premise is that the country’s domestic market is sufficiently huge and the industrial system complete.

Second, no matter whether a domestic trade network or an international trade network, economies with better macroeconomic performance usually have higher nestedness. We believe that it is the relatively mature trade mechanisms that bring with them economic benefits and avoided risks. Hence, both domestic and international markets are equally important for a country’s economic development. How to better connect and utilize them is the key for countries to gain new advantages in international cooperation and competition.

Third, the ETN-NODF and ITN-NODF of the U. S. show a negative correlation with its GDP. In recent years, the U.S. has integrated a large amount of capital into the highly lucrative consumption side and shifted low-end manufacturing to countries with cheap labor costs, thus leading to the advent of manufacturing hollowing-out. Combined with industrial shocks from many developed (e.g., Germany and Japan) and developing countries (e.g., China), the international trade cycle does not seem to be contributing to the macroeconomic performance of the U.S. as expected. Such an industrial layout of the U.S. undermines its stability when encountering the rare but severe systematic risk. For instance, it is impressive that the White House could not obtain enough prevention and control supplies at the beginning of the COVID-19 pandemic.

### 6.2. Regression Model

To describe the quantitative relationship between GDP and the NODF-based indicators accurately, this paper applies regression analysis on these variables. First, a mixed effect regression model was established by taking the GDP of each country as the dependent variable and DTN-NODF, ETN-NODF and ITN-NODF as the independent variables, as shown in Table 1.

The correlation coefficients between the three independent variables were examined to check the existence of multicollinearity problems in the above model, so as to avoid spurious regression and ensure the validity of the model. The results show that the two variables, ETN-NODF and ITN-NODF, are significantly correlated, with correlation coefficients as high as 0.8608 (0.2631 for DTN-NODF and ETN-NODF, and 0.3289 for DTN-NODF and ITN-NODF).

To avoid multicollinearity problems in the following regression, Principal Component Analysis (PCA) was performed between ETN-NODF and ITN-NODF to investigate the components that constitute their covariance and ensure the orthogonality of the independent variables [45]. The ranked components with their loadings are listed in Table 2, revealing that both the KMO and the SMC confirm the correctness of PCA. The first principal components of PCA results are retained in this paper. Meanwhile, as shown in Appendix D, Table A5, we used a Ridge Regression to find the penalization term and found that the coefficients and conclusions are robust. We also provided the results of Fixed Effects model (FE), Random Effects model (RE) and Least Square Dummy Variable model (LSDV). As shown in Appendix D, Table A6, Table A7 and Table A8, we concluded that the pooled regression, i.e., Mixed Effect Regression model with PCA is the optimal solution (Appendix D).

As shown in Table 3, the model exhibits a relatively good fit with a large $R^{2}\;$ and all the independent variables (DTN-NODF and component variables) pass the *p*-value test at the significance level of 0.01.

From the above results, DTN-NODF displays a weak negative correlation with GDP. It is believed that an excessively nested domestic trade network may hinder a country’s economic development. Although a highly nested industrial layout can enhance the stability of the production system, it can also bring about problems such as lack of effective competition, path dependence in innovation and blocked channels for international cooperation, thereby hampering the country’s macroeconomic performance. On the other hand, ETN-NODF and ITN-NODF show a significant positive correlation with GDP. This can be attributed to the fact that for those who actively participates in the international trade cycle, they can complement each other by their own advantages and efficiently leverage resources in the international market, which leads to domestic socioeconomic development.

Besides, the regression model indicates that the positive effect of ETN-NODF on GDP is about two times greater than that of ITN-NODF. This is also a self-evident phenomenon. Larger ETN-NODF means a higher degree of nestedness in the export trade network structure, more orderly export mechanisms and more stable relevant channels, which together help to increase the trade surplus and boost domestic economic growth. At the same time, export growth drives import growth and secures the source of raw materials for the normal functioning of a country’s production system. As a result, this promotes the healthy development of the import trade network in turn, along with the increase of ITN-NODF. However, compared to the export trade network that can be unstoppably expanded, the expansion of the import trade network is limited by the relatively homogenous source of raw materials. That is why ETN-NODF is higher in value than ITN-NODF.

## 7. Conclusions

The vertical international division of labor and the continuous development of the global production network have played an unprecedented role in promoting the economy and trade of all countries in the world. The dynamic exchange of resources across the world is also an important guarantee for the stable and orderly progress of the GVC network. Through static and dynamic analysis of the generalist degree of the industrial sectors, the phenomenon is found that most of the generalist sectors come from more developed economies, which are in the most closely nested areas, and their internal competition is fierce. Due to the rapid increase in the generalist degree of China’s manufacturing industry, the manufacturing and services sectors in Japan and Taiwan have shown a downward trend, which has paved the way for changes in the global supply chain pattern. These advanced economies, as well as their industrial sectors, have also played a decisive role in maintaining the stability of the GVC network and promoting the process of global economic integration. In addition, there is still a lot of room for optimization of the industrial layout in the GVC. Encouraging specialist sectors to actively integrate into a global trade system is an effective way to realize this.

## Figures and Tables

**Table 1:** Results of the Mixed Effect Regression Model.

Variables	Coef.	Robust Std. Err	t	*p*	95% Confidence Interval
DTN-NODF	−9.49	2.64	−3.60	0.000	[−14.67, −4.32]
ETN-NODF	44.27	3.88	11.40	0.000	[36.65, 51.90]
ITN-NODF	21.03	6.88	3.06	0.002	[7.54, 34.52]
Intercept Term	71.97	180.36	0.40	0.690	[−281.95, 425.89]
$R^{2}$ (adjusted)	0.411		Root MSE	867.68	

**Table 2:** Principal Component Analysis of ETN-NODF and ITN-NODF.

Component	Eigenvalue	Difference	Proportion	Cumulative	KMO	SMC
ETN-NODF	233.326	220.745	0.9488	0.9488	0.9999	0.7410
ITN-NODF	12.581	-	0.0512	1.0000	0.9999	0.7410

**Table 3:** Results of Mixed Effect Regression Model after PCA.

Variables	Coef.	Robust Std. Err	t	*p*	95% Confidence Interval
DTN-NODF	−9.63	2.60	−3.71	0.000	[−14.72, −4.54]
Comp.	48.99	1.83	26.74	0.000	[45.39, 52.58]
Intercept Term	74.09	180.14	0.41	0.681	[−279.40, 427.57]
$R^{2}$ (adjusted)	0.411		Root MSE	867.3	

Notes: Comp. = 0.8824ETN-NODF + 0.4705ITN-NODF.

**Figure A1:**
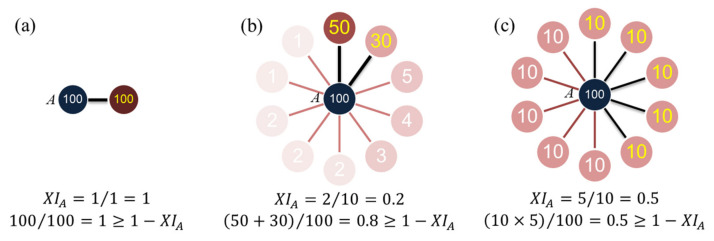
Three possible situations in the application of XIFA algorithm. (**a**) The source node has only one weighted edge connected to it, and 100% of its strength is allocated on it; (**b**) Top 20% of weighted edges carry 80% of the strength of source node. (**c**) Any 50% of weighted edges carry 50% of the strength of source node.

**Figure A2:**
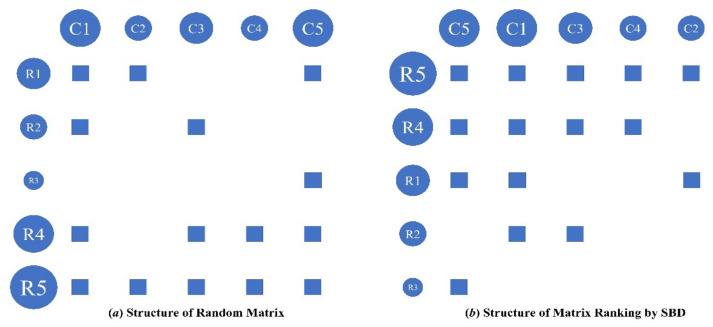
Sorting Adjacency Matrix Based on SBD Algorithm. This is a schematic diagram of the process of ordering nodes by degree. The solid blue circles represent each industrial sector, the rows represent the upstream industrial sectors, the columns represent the downstream industry sectors, the blue squares represent the existence of interdependence between upstream and downstream industries, and the size of the solid circles is proportional to the node’s degree.

**Figure A3:**
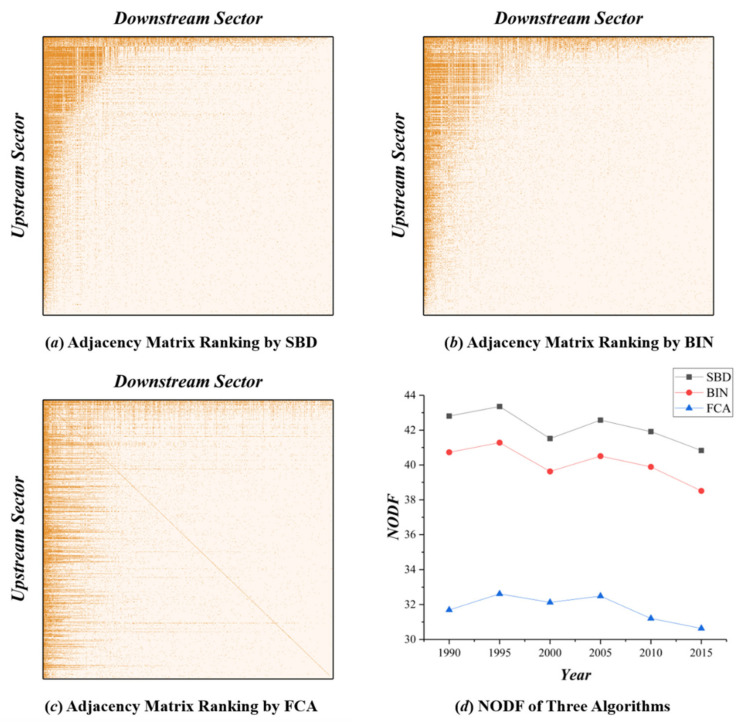
Sorting Adjacency Matrix of GVC Network Based on Three Algorithms and Its Corresponding NODF Variation Trend. (**a**–**c**) are the adjacency matrix ranking results obtained according to the SBD, BIN, and FCA algorithms, respectively. Where the vertical axis represents the upstream sector and the horizontal axis represents the downstream sector, and each non-empty position reflects the transfer of intermediate products from the upstream sector to the downstream sector. This input–output relationship between industrial sectors resembles predation in an ecosystem: the upstream sector, as the provider of energy (products and services), can be regarded as the prey; the downstream sector, as the consumer of energy (products and services), can be regarded as the predator, and each industry sector plays dual role in the industrial ecosystem.

## Data Availability

We signed a confidentiality agreement with the transportation company who provided us with the data used in this work. Hence the data will not be shared.

## Appendix A. Basic Information about Eora26

**Table A1:** Countries’ Names and Their Abbreviations of Eora26.

No.	Abbr.	Country	No.	Abbr.	Country
1	AFG	Afghanistan	96	LSO	Lesotho
2	ALB	Albania	97	LBR	Liberia
3	DZA	Algeria	98	LBY	Libya
4	AND	Andorra	99	LIE	Liechtenstein
5	AGO	Angola	100	LTU	Lithuania
6	ATG	Antigua	101	LUX	Luxembourg
7	ARG	Argentina	102	MAC	Macao SAR
8	ARM	Armenia	103	MDG	Madagascar
9	ABW	Aruba	104	MWI	Malawi
10	AUS	Australia	105	MYS	Malaysia
11	AUT	Austria	106	MDV	Maldives
12	AZE	Azerbaijan	107	MLI	Mali
13	BHS	Bahamas	108	MLT	Malta
14	BHR	Bahrain	109	MRT	Mauritania
15	BGD	Bangladesh	110	MUS	Mauritius
16	BRB	Barbados	111	MEX	Mexico
17	BLR	Belarus	112	MCO	Monaco
18	BEL	Belgium	113	MNG	Mongolia
19	BLZ	Belize	114	MNE	Montenegro
20	BEN	Benin	115	MAR	Morocco
21	BMU	Bermuda	116	MOZ	Mozambique
22	BTN	Bhutan	117	MMR	Myanmar
23	BOL	Bolivia	118	NAM	Namibia
24	BIH	Bosnia and Herzegovina	119	NPL	Nepal
25	BWA	Botswana	120	NLD	Netherlands
26	BRA	Brazil	121	ANT	Netherlands Antilles
27	VGB	British Virgin Islands	122	NCL	New Caledonia
28	BRN	Brunei	123	NZL	New Zealand
29	BGR	Bulgaria	124	NIC	Nicaragua
30	BFA	Burkina Faso	125	NER	Niger
31	BDI	Burundi	126	NGA	Nigeria
32	KHM	Cambodia	127	NOR	Norway
33	CMR	Cameroon	128	PSE	Gaza Strip
34	CAN	Canada	129	OMN	Oman
35	CPV	Cape Verde	130	PAK	Pakistan
36	CYM	Cayman Islands	131	PAN	Panama
37	CAF	Central African Republic	132	PNG	Papua New Guinea
38	TCD	Chad	133	PRY	Paraguay
39	CHL	Chile	134	PER	Peru
40	CHN	China	135	PHL	Philippines
41	COL	Colombia	136	POL	Poland
42	COG	Congo	137	PRT	Portugal
43	CRI	Costa Rica	138	QAT	Qatar
44	HRV	Croatia	139	KOR	South Korea
45	CUB	Cuba	140	MDA	Moldova
46	CYP	Cyprus	141	ROU	Romania
47	CZE	Czech Republic	142	RUS	Russia
48	CIV	Cote d’Ivoire	143	RWA	Rwanda
49	PRK	North Korea	144	WSM	Samoa
50	COD	DR Congo	145	SMR	San Marino
51	DNK	Denmark	146	STP	Sao Tome and Principe
52	DJI	Djibouti	147	SAU	Saudi Arabia
53	DOM	Dominican Republic	148	SEN	Senegal
54	ECU	Ecuador	149	SRB	Serbia
55	EGY	Egypt	150	SYC	Seychelles
56	SLV	El Salvador	151	SLE	Sierra Leone
57	ERI	Eritrea	152	SGP	Singapore
58	EST	Estonia	153	SVK	Slovakia
59	ETH	Ethiopia	154	SVN	Slovenia
60	FJI	Fiji	155	SOM	Somalia
61	FIN	Finland	156	ZAF	South Africa
62	FRA	France	157	SDS	South Sudan
63	PYF	French Polynesia	158	ESP	Spain
64	GAB	Gabon	159	LKA	Sri Lanka
65	GMB	Gambia	160	SUD	Sudan
66	GEO	Georgia	161	SUR	Suriname
67	DEU	Germany	162	SWZ	Swaziland
68	GHA	Ghana	163	SWE	Sweden
69	GRC	Greece	164	CHE	Switzerland
70	GRL	Greenland	165	SYR	Syria
71	GTM	Guatemala	166	TWN	Taiwan
72	GIN	Guinea	167	TJK	Tajikistan
73	GUY	Guyana	168	THA	Thailand
74	HTI	Haiti	169	MKD	TFYR Macedonia
75	HND	Honduras	170	TGO	Togo
76	HKG	Hong Kong	171	TTO	Trinidad and Tobago
77	HUN	Hungary	172	TUN	Tunisia
78	ISL	Iceland	173	TUR	Turkey
79	IND	India	174	TKM	Turkmenistan
80	IDN	Indonesia	175	USR	Former USSR
81	IRN	Iran	176	UGA	Uganda
82	IRQ	Iraq	177	UKR	Ukraine
83	IRL	Ireland	178	ARE	United Arab Emirates
84	ISR	Israel	179	GBR	United Kingdom
85	ITA	Italy	180	TZA	Tanzania
86	JAM	Jamaica	181	USA	United States
87	JPN	Japan	182	URY	Uruguay
88	JOR	Jordan	183	UZB	Uzbekistan
89	KAZ	Kazakhstan	184	VUT	Vanuatu
90	KEN	Kenya	185	VEN	Venezuela
91	KWT	Kuwait	186	VNM	Viet Nam
92	KGZ	Kyrgyzstan	187	YEM	Yemen
93	LAO	Laos	188	ZMB	Zambia
94	LVA	Latvia	189	ZWE	Zimbabwe
95	LBN	Lebanon			

**Table A2:** Eora26 Industry Code Description.

No.	Abbr.	Industrial Sector
1	S1	Agriculture
2	S2	Fishing
3	S3	Mining and Quarrying
4	S4	Food & Beverages
5	S5	Textiles and Wearing Apparel
6	S6	Wood and Paper
7	S7	Petroleum, Chemical and Non-Metallic Mineral Products
8	S8	Metal Products
9	S9	Electrical and Machinery
10	S10	Transport Equipment
11	S11	Other Manufacturing
12	S12	Recycling
13	S13	Electricity, Gas and Water
14	S14	Construction
15	S15	Maintenance and Repair
16	S16	Wholesale Trade
17	S17	Retail Trade
18	S18	Hotels and Restaurants
19	S19	Transport
20	S20	Post and Telecommunications
21	S21	Financial Intermediation and Business Activities
22	S22	Public Administration
23	S23	Education, Health and Other Services
24	S24	Private Households
25	S25	Others
26	S26	Re-export & Re-import

**Table A3:** Four-Sector Categories in Eora26.

Category	Sectors
Agriculture (SC1)	Agriculture; Fishing
Mining (SC2)	Mining and Quarrying
Manufacturing (SC3)	Food & Beverages; Textiles and Wearing Apparel; Wood and Paper; Petroleum, Chemical and Non-Metallic Mineral Products; Metal Products; Electrical and Machinery; Transport Equipment; Other Manufacturing; Recycling; Electricity, Gas and Water; Construction
Services (SC4)	Maintenance and Repair; Wholesale Trade; Retail Trade; Hotels and Restaurants; Transport; Post and Telecommunications; Financial Intermediation and Business Activities; Public Administration; Education, Health and Other Services; Private Households; Others; Re-export & Re-import

## Appendix B. Network Pruning

The GIVCNBG model is a very dense weighted network, and there is a more or less value flow between each upstream sector and downstream sector. Therefore, before analyzing the nested structure, the network needs to be pruned to extract the backbone. In order to find an effectual way to extract the backbone of weighted network, two questions are proposed. The first question is that the information content of network should be retained as much as possible with the decline of number of edges (Q1). In other words, the nature of network pruning is a trade-off between the number of retained edges and the gross of the retained weights. The second question is that only the truly important edges linking nodes are worthy to be retained during downsizing of the network (Q2). Sometimes, a weighted edge is numerically insignificant but functionally significant, and reckless deletion will result in a useless broken structure.

It is common in the weighted networks that, even at the local level defined by edges linking to a given node, only a few of those edges carry a disproportionate fraction of its strength, and the remaining edges take a very small percentage that is left. Enlightened by H-Index, the Pareto Principle and the idea of the Disparity Filter proposed by Vespignani, we present a novel heuristic algorithm to effectively prune the dense and weighted GIVCN model, which is named X-Index Filtering Algorithm (XIFA).

As we all know, Hirsch proposed that a scientist has index h if h of his or her $N_{p}$ paper have at least h citations each and the other $\left(N_{p}-h\right)$ papers have ≤h citations each. Obviously, this mixed quantitative index takes both the quantity and quality of papers into account, which can be used as an algorithm framework to solve Q1 of this paper by weighing the pros and cons, namely the number and weights of edges. According to the Pareto principle, it makes sense that 80% of consequences come from 20% of the causes, asserting an unequal relationship between inputs and outputs. We hence assume that a minority of edges hold the most weight in the network, which are regarded as the so-called important edges as mentioned in Q2.

Therefore, the core idea of XIFA is as follows: An industrial sector has backward index x ($XI^{B}$)/forward index x ($XI^{F}$) if top x percent of its relations to all upstream/downstream sectors occupy at least $\left(1-x\%\right)$ of its total input/output amount of intermediate goods. The formula deduction and practical calculation process is shown in Figure A1. According to the induction hypothesis, the X-index of a node is measured by the number of crucial edges with big to small weights, and a smaller X-index value indicates that the edge weight distribution of this node is more heterogeneous. In the directed networks, the XIFA is initiated by removing all indegree edges of each node that are insufficient to show the local influence, and the pruning process is then repeated for the outdegree edges. It should be noted that the existence of some edges tended to be overlooked, for instance, certain edges are important in terms of indegree but unimportant in terms of outdegree and vice versa, and one more rule is hence set, that is, the weighted edges that are worthy to be reserved in either direction will stay.

A special subgraph $\overset{´}{G}=\left(O,P,\overset{´}{E},\overset{´}{W}\right)$ is extracted from the GIVCNBG model based on XIFA, which is named the GIVCNBG-FE model (FE refers to filtering edges). The remaining MRIO relations are different from those deleted depending on how heterogeneous the industrial sectors’ inputs or outputs are all over the world. We can assume that around 20% of the most important input or output relations of a given sector are supposed to cover 80% of its input or output amount of intermediate goods, which addresses Q1 favorably.

**Table A4:** The Procedure of Pruning GIVCN Model Based on XIFA.

Procedure	Column Deletion of Input Relations	Row Deletion of Output Relations
Network	$W={\left(w_{ij}\right)}_{N\times N}\cdot \;$ $i,j\in \left[1,N\right]$	
Refactoring	${\overset{↼}{w}}_{1}=desecend{\left(w_{11},w_{21,}\cdots ,w_{N1}\right)}^{T}$ ${\overset{↼}{w}}_{2}=descend{\left(w_{12},w_{22,}\cdots ,w_{N2}\right)}^{T}$ ⋯ ${\overset{↼}{w}}_{N}=descend{\left(w_{1N},w_{2N,}\cdots ,w_{NN}\right)}^{T}$ $\overset{↼}{W}=\left({\overset{↼}{w}}_{1},{\overset{↼}{w}}_{2},\cdots ,{\overset{↼}{w}}_{N}\right)={\left({\overset{↼}{w}}_{sj}\right)}_{N\times N}$ $s,j\in \left[1,N\right]$	${\vec{w}}_{1}=desecend\left(w_{11},w_{12,}\cdots ,w_{1N}\right)$ ${\vec{w}}_{2}=descend\left(w_{21},w_{22,}\cdots ,w_{2N}\right)$ ⋯ ${\vec{w}}_{N}=descend\left(w_{N1},w_{N2,}\cdots ,w_{NN}\right)$ $\vec{W}=\left(\begin{array}{c} {\vec{w}}_{1}\\ {\vec{w}}_{2}\\ \cdots \\ {\vec{w}}_{N} \end{array}\right)={\left({\vec{w}}_{it}\right)}_{N\times N}$ $i,t\in \left[1,N\right]$
Conditions	$\forall a_{1},a_{2},\cdots ,a_{j}\in \left[1,N\right]$ $\left\{\begin{array}{c} \frac{\sum_{s=1}^{a_{j}}{\overset{↼}{w}}_{sj}}{\sum_{s=1}^{N}{\overset{↼}{w}}_{sj}}\geq 1-\frac{a_{j}}{N}\\ \frac{\sum_{s=1}^{a_{j}-1}{\overset{↼}{w}}_{sj}}{\sum_{s=1}^{N}{\overset{↼}{w}}_{sj}}<1-\frac{a_{j}-1}{N} \end{array}\right.$	$\forall b_{1},b_{2},\cdots ,b_{i}\in \left[1,N\right]$ $\left\{\begin{array}{c} \frac{\sum_{t=1}^{b_{i}}{\vec{w}}_{it}}{\sum_{t=1}^{N}{\vec{w}}_{it}}\geq 1-\frac{b_{i}}{N}\\ \frac{\sum_{t=1}^{b_{i}-1}{\vec{w}}_{it}}{\sum_{t=1}^{N}{\vec{w}}_{it}}<1-\frac{b_{i}-1}{N} \end{array}\right.$
Definition	$XI_{j}^{B}=\frac{a_{j}}{N}$ $XI^{B}={\left(XI_{j}^{B}\right)}_{N\times 1}$	$XI_{i}^{F}=\frac{b_{i}}{N}$ $XI^{F}={\left(XI_{i}^{F}\right)}_{N\times 1}$
Pruning	${\overset{\leftarrow }{w}}_{ij}=\left\{\begin{array}{ccc} w_{ij} & , & w_{ij}={\overset{↼}{w}}_{sj}\;and\;s\leq a_{j}\\ 0 & , & otherwise \end{array}\right.$	${\vec{w}}_{ij}=\left\{\begin{array}{ccc} w_{ij} & , & w_{ij}={\vec{w}}_{it}\;and\;t\leq b_{i}\\ 0 & , & otherwise \end{array}\right.$
Merging	${\overset{↔}{w}}_{ij}=\left\{\begin{array}{ccc} w_{ij} & , & {\overset{\leftarrow }{w}}_{ij}\neq 0\;or\;{\vec{w}}_{ij}\neq 0\\ 0 & , & otherwise \end{array}\right.$	
Result	$\overset{↔}{W}={\left({\overset{↔}{w}}_{ij}\right)}_{N\times N}$	

The XIFA algorithm is a mixed quantitative indicator, which takes into account the scope and intensity of the influence of the industrial sector, extracting the main topology of the industrial network according to the heterogeneity of input and output among the industrial sectors. From the perspective of complex networks, for nodes with strong edge weight heterogeneity, only a small number of connections with extremely large weights are needed to be kept, while for nodes with weak edge weight heterogeneity, the number of connections needed to be kept is greater but not more than 50%.

From the perspective of industrial economics, the integrity of the GVC must be considered. In consequence, a method of pruning separately from the aspect of output and input, then integrating, was finally adopted. This prevents certain industrial sectors from being too marginalized from the GVC.

Moreover, overlapping two subnetworks via the input relations (columns) and output relations (rows) pruning process is only a partial solution to Q2 because this pruning method is still based on the relations with adjacent nodes rather than global information. Therefore, to solve Q1 and Q2 at the same time a hybrid strategy needs to be adopted, a specific part in our other paper “*Extracting the Backbone of Global Value Chain from High-Dimensional Multi-Region Input–Output Network*”. For the nested structure to be studied in this article, the GIVCNBG-FE model has extracted enough GVC network topology information.

## Appendix C. Sorting Algorithms

1. SBD Algorithm

Sorted by Degree, or the SBD Algorithm, based on the concept of nestedness, sorts the adjacency matrix according to the degree of the network node (see Figure A2). It is prescribed in the nested structure that the neighborhood of lower-degree nodes is a subset of the neighborhood of higher degree nodes. Accordingly, the SBD algorithm basically rearranges the adjacency matrix’s rows and columns in the descending order of the node’s degree from top to bottom and from left to right respectively. In the rearranged network adjacency matrix, the topmost upstream sector boasts the largest number of downstream sectors, while the leftmost downstream sector boasts the largest number of the most upstream sectors.

2. NTC Algorithm

The Nestedness Temperature Calculator (NTC Algorithm) is a thermodynamics-based algorithm proposed by Atmar, focusing on the degree of disorder of the measurement matrix [46]. The nested structure features ordered arrangement of nodes, therefore, the more disordered the adjacency matrix, the higher its temperature, and the lower the level of nestedness. The NTC Algorithm lines out a perfect nested region at the top left of the adjacency matrix, and the unexpected absence of any element above the line and the unexpected appearance of any element below the line results in an increase in the temperature of the adjacency matrix.

3. BIN Algorithm

BINMATNEST Algorithm (the BIN Algorithm) was proposed by Rodrı’guez-Girone´s et al., which compensates for the shortcomings of the NTC, e.g., the non-uniqueness of the perfect order line and the inadequacy of null model selection [47]. In design, the BIN Algorithm is a genetic algorithm that minimizes the matrix temperature by rearranging the rows and columns. It first generates some alternative solutions and then lets the well-performing matrix generate “Offspring”, thus, iteratively filtering out the best-performing solution. Unlike the NTC algorithm, the BIN algorithm is capable of screening out the optimal matrix with lower temperature. The matrix reaches the lowest temperature after reordering and is therefore more well-organized, with stabler connections between industrial sectors concentrated at the upper left corner. Under the circumstances, the NTC algorithm is not worth discussing anymore.

4. FCA Algorithm

The Fitness-Complexity Algorithm (FCA Algorithm) applies a nonlinear iterative method originally designed to measure economic complexity [48]. The mechanism is that the higher the fitness of a country, the higher its productive capacity or competitiveness; the higher the complexity of a certain product, then, the higher the productive capacity required from other countries producing that product. In the adjacency matrix of the country-product network, the rows represent countries and the columns the export products. After reordering the matrix in the descending order of fitness from top to bottom and product complexity from left to right, the new matrix exhibits distinct nestedness. The FCA therefore can be used to explore the maximum nestedness of a network.

We apply the GIVCNBG-FE-Eora26SC4 model to calculate the nestedness of the GVC network at intervals of five years, and compare the sorting results of the SBD, BIN and FCA algorithms and their NODF metrics, as shown in Figure A3.

The SBD algorithm sorts the adjacency matrix in a way that most of the non-zero elements are clustered at the upper left corner. The results obtained by the BIN algorithm resemble that of the SBD algorithm, with the upper left corner being sparser. The results obtained by the FCA algorithm are computationally weaker than the other two algorithms. Given the above analysis (see Figure A3d), the SBD algorithm is thus used in this paper to sort the nested structure of the adjacency matrix. The overall smooth NODF values indicate temporal stability of the nested structure, which means the topology of the GVC network does not change drastically during a normal economic cycle.

## Appendix D. Complementary Econometric Analysis

**Table A5:** Results of the Ridge Regression Model.

Variables	Coef.	Robust Std. Err	t	*p*	95% Confidence Interval
DTN-NODF	−9.49	2.64	−3.60	0.000 ***	[−14.67, −4.32]
ETN-NODF	44.27	3.88	11.40	0.000 ***	[36.65, 51.90]
ITN-NODF	21.03	6.88	3.06	0.002 **	[7.54, 34.52]
Intercept Term	71.97	180.36	0.40	0.690	[−281.95, 425.89]
$R^{2}\;$(adjusted)	0.411		Root MSE	867.68	

Notes: “***” represents *p* < 0.001, “**” represents *p* < 0.01, and “*” represents *p* < 0.05. There is basically no difference between the coefficient of ridge regression and the mixed regression with PCA. For example, the coefficients of DTN-NODF and ETN-NODF in ridge regression are −9.49 and 44.27, and the mixed regression are −9.63, 48.99 × 0.8824 = 43.23. Thus, the regression coefficients and related conclusions of the mixed regression model used in this paper are robust.

**Table A6:** Results of the FE Model.

Variables	Coef.	Robust Std. Err	t	*p*	95% Confidence Interval
DTN-NODF	0.40	1.99	0.20	0.840	[−3.52, 4.33]
Comp.	43.27	45.56	0.95	0.343	[−46.62, 133.17]
Intercept Term	−538.56	712.27	−0.76	0.451	[−1943.92, 866.80]

Notes: All coefficients in the fixed effects model failed the test.

**Table A7:** Results of the LSDV Model.

Variables	Coef.	Robust Std. Err	t	*p*	95% Confidence Interval
DTN-NODF	0.40	2.19	0.18	0.854	[−3.91, 4.72]
Comp.	43.27	50.14	0.86	0.389	[−55.65, 142.20]
country					
AFG	346.87	353.84	0.98	0.328	[−351.2987, 1045.033]
AGO	281.05	257.66	1.09	0.277	[−227.3288, 789.4262]
ALB	216.71	218.33	0.99	0.322	[−214.0759, 647.4973]
AND	236.70	238.52	0.99	0.322	[−233.9195, 707.3123]
ARE	422.05	276.65	1.53	0.129	[−123.7993, 967.9091]
ARG	−346.32	749.44	−0.46	0.645	[−1825.031, 1132.375]
ARM	214.61	231.28	0.93	0.355	[−241.7304, 670.9555]
ATG	202.95	188.93	1.07	0.284	[−169.8275, 575.7282]
AUS	−477.86	1384.80	−0.35	0.73	[−3210.196, 2254.469]
AUT	−134.15	501.67	−0.27	0.789	[−1123.986, 855.6903]
AZE	211.72	206.19	1.03	0.306	[−195.0985, 618.5441]
BDI	197.72	179.91	1.1	0.273	[−157.2522, 552.6847]
BEL	−1179.98	1749.33	−0.67	0.501	[−4631.553, 2271.592]
BEN	266.94	276.91	0.96	0.336	[−279.4249, 813.3128]
BFA	197.92	209.44	0.94	0.346	[−215.324, 611.1616]
BGD	463.47	441.16	1.05	0.295	[−406.9706, 1333.907]
BGR	322.55	330.76	0.98	0.331	[−330.0761, 975.1704]
BHR	321.21	341.12	0.94	0.348	[−351.8472, 994.2654]
BHS	263.53	275.51	0.96	0.34	[−280.0784, 807.1395]
BIH	303.62	310.98	0.98	0.33	[−309.9727, 917.2204]
BLR	109.25	102.04	1.07	0.286	[−92.07386, 310.582]
BLZ	142.7	156.80	0.91	0.364	[−166.6857, 452.0805]
BMU	163.46	161.68	1.01	0.313	[−155.5533, 482.4662]
BOL	80.53	84.62	0.95	0.343	[−86.4349, 247.4872]
BRA	527.65	715.03	0.74	0.462	[−883.1721, 1938.471]
BRB	282.83	291.34	0.97	0.333	[−292.0029, 857.6546]
BRN	334.04	351.24	0.95	0.343	[−358.9823, 1027.066]
BTN	79.27	88.60	0.89	0.372	[−95.53579, 254.075]
BWA	285.04	305.34	0.93	0.352	[−317.4236, 887.4988]
CAF	265.57	260.87	1.02	0.31	[−249.161, 780.2916]
CAN	770.19	341.90	2.25	0.025 *	[95.58298, 1444.791]
CHE	−909.9	1534.75	−0.59	0.554	[−3938.092, 2118.301]
CHL	−60.5	228.66	−0.26	0.792	[−511.6715, 390.6751]
CHN	1503.19	2459.50	0.61	0.542	[−3349.617, 6355.998]
CIV	346.24	367.63	0.94	0.348	[−379.13, 1071.614]
CMR	360.41	378.99	0.95	0.343	[−387.3771, 1108.196]
COD	265.26	265.10	1	0.318	[−257.8036, 788.316]
COG	290.82	307.71	0.95	0.346	[−316.319, 897.9642]
COL	81.02	76.04	1.07	0.288	[−69.00603, 231.0458]
CPV	219	208.95	1.05	0.296	[−193.2711, 631.2752]
CRI	390.24	414.36	0.94	0.348	[−427.3227, 1207.812]
CUB	381.23	369.91	1.03	0.304	[−348.6375, 1111.096]
CYP	342.62	366.80	0.93	0.352	[−381.1153, 1066.35]
CZE	−172.23	317.34	−0.54	0.588	[−798.369, 453.9041]
DEU	198.25	2855.59	0.07	0.945	[−5436.072, 5832.577]
DJI	163.01	138.96	1.17	0.242	[−111.1765, 437.1888]
DNK	−703.72	1088.11	−0.65	0.519	[−2850.657, 1443.218]
DOM	416.84	427.58	0.97	0.331	[−426.8171, 1260.502]
DZA	408.18	350.16	1.17	0.245	[−282.7166, 1099.085]
ECU	−118.3	175.10	−0.68	0.5	[−463.7915, 227.1998]
EGY	511.76	410.13	1.25	0.214	[−297.4581, 1320.971]
ERI	235.58	222.08	1.06	0.29	[−202.6103, 673.7649]
ESP	−609.87	1790.20	−0.34	0.734	[−4142.082, 2922.346]
EST	−48.19	69.35	−0.69	0.488	[−185.0211, 88.64208]
ETH	−107.18	134.83	−0.79	0.428	[−373.2024, 158.8407]
FIN	−20.37	245.53	−0.08	0.934	[−504.8219, 464.0865]
FJI	276.83	299.98	0.92	0.357	[−315.0676, 868.72]
FRA	−1.51	2245.24	0	0.999	[−4431.562, 4428.534]
GAB	314.42	332.34	0.95	0.345	[−341.3062, 970.1512]
GBR	−1022.55	3512.17	−0.29	0.771	[−7952.356, 5907.252]
GEO	−178.73	200.24	−0.89	0.373	[−573.8264, 216.3583]
GHA	323.03	336.75	0.96	0.339	[−341.3976, 987.4673]
GIN	264.45	280.28	0.94	0.347	[−288.5671, 817.4742]
GMB	203.94	205.10	0.99	0.321	[−200.7466, 608.6262]
GRC	100.46	91.75	1.09	0.275	[−80.57459, 281.495]
GRL	375.57	432.99	0.87	0.387	[−478.7589, 1229.897]
GTM	400.99	420.56	0.95	0.342	[−428.8063, 1230.781]
GUY	403.69	444.55	0.91	0.365	[−473.4424, 1280.816]
HKG	−172.88	417.98	−0.41	0.68	[−997.5889, 651.824]
HND	351.7	384.12	0.92	0.361	[−406.2073, 1109.612]
HRV	393.84	411.64	0.96	0.34	[−418.3654, 1206.055]
HTI	322.38	338.26	0.95	0.342	[−345.0331, 989.8003]
HUN	−39.7	165.92	−0.24	0.811	[−367.0765, 287.6826]
IDN	−204.47	695.67	−0.29	0.769	[−1577.078, 1168.141]
IND	−405.95	1554.43	−0.26	0.794	[−3472.961, 2661.06]
IRL	61.82	107.64	0.57	0.566	[−150.5622, 274.2033]
IRN	−367.70	704.14	−0.52	0.602	[−1757.034, 1021.625]
IRQ	287.09	163.82	1.75	0.081	[−36.13326, 610.32]
ISL	336.34	362.54	0.93	0.355	[−378.9751, 1051.662]
ISR	−403.11	647.93	−0.62	0.535	[−1681.534, 875.3045]
ITA	−358.7	2247.07	−0.16	0.873	[−4792.36, 4074.952]
JAM	351.8	378.13	0.93	0.353	[−394.2818, 1097.891]
JOR	325.8	359.43	0.91	0.366	[−383.3841, 1034.987]
JPN	2047.56	3094.29	0.66	0.509	[−4057.734, 8152.856]
KAZ	−244.62	362.13	−0.68	0.5	[−959.1294, 469.8803]
KEN	−197.97	244.35	−0.81	0.419	[−680.0901, 284.1591]
KGZ	−455.86	512.61	−0.89	0.375	[−1467.283, 555.5592]
KHM	289.57	316.28	0.92	0.361	[−334.4798, 913.6205]
KOR	−313.24	1286.85	−0.24	0.808	[−2852.31, 2225.822]
KWT	−154.41	235.90	−0.65	0.514	[−619.8692, 311.0498]
LAO	270.07	289.50	0.93	0.352	[−301.147, 841.2839]
LBN	323.88	326.75	0.99	0.323	[−320.815, 968.5826]
LBR	184.38	192.94	0.96	0.341	[−196.318, 565.0694]
LBY	365.12	357.11	1.02	0.308	[−339.4948, 1069.737]
LIE	302.74	309.76	0.98	0.33	[−308.4337, 913.9225]
LKA	355.78	375.62	0.95	0.345	[−385.3557, 1096.923]
LSO	134.13	131.54	1.02	0.309	[−125.4121, 393.6786]
LTU	−77.57	124.47	−0.62	0.534	[−323.162, 168.0136]
LUX	3.05	33.70	0.09	0.928	[−63.45578, 69.54905]
LVA	−70.4	97.66	−0.72	0.472	[−263.0863, 122.2896]
MAC	235.37	247.68	0.95	0.343	[−253.3254, 724.0649]
MAR	426.86	415.58	1.03	0.306	[−393.1085, 1246.832]
MCO	228.67	228.95	1	0.319	[−223.0632, 680.4089]
MDA	26.78	37.72	0.71	0.479	[−47.63299, 101.1987]
MDG	293.64	318.76	0.92	0.358	[−335.2989, 922.5689]
MDV	88.83	87.04	1.02	0.309	[−82.90518, 260.5718]
MEX	374.19	398.69	0.94	0.349	[−412.467, 1160.84]
MKD	−157.69	179.44	−0.88	0.381	[−511.7428, 196.361]
MLI	275.04	271.87	1.01	0.313	[−261.3773, 811.4663]
MLT	−125.21	149.46	−0.84	0.403	[−420.1182, 169.6895]
MMR	456.09	479.59	0.95	0.343	[−490.168, 1402.357]
MNE	32.86	22.77	1.44	0.151	[−12.06697, 77.78323]
MNG	209.38	216.84	0.97	0.336	[−218.4693, 637.2248]
MOZ	277.65	287.27	0.97	0.335	[−289.1506, 844.4498]
MRT	136.57	144.52	0.94	0.346	[−148.5807, 421.7112]
MUS	−221.78	246.34	−0.9	0.369	[−707.8259, 264.269]
MWI	265.54	286.68	0.93	0.356	[−300.1154, 831.1903]
MYS	−432.16	665.46	−0.65	0.517	[−1745.172, 880.8547]
NAM	282.94	314.23	0.9	0.369	[−337.0612, 902.9311]
NCL	285.79	312.88	0.91	0.362	[−331.5583, 903.1301]
NER	211.67	211.31	1	0.318	[−205.2679, 628.5985]
NGA	462.42	328.72	1.41	0.161	[−186.1661, 1111.009]
NIC	298.84	323.29	0.92	0.357	[−339.0496, 936.7254]
NLD	−1257.85	2128.45	−0.59	0.555	[−5457.455, 2941.764]
NOR	171.77	144.10	1.19	0.235	[−112.5632, 456.0975]
NPL	322.12	342.61	0.94	0.348	[−353.8727, 998.1114]
NZL	−324.92	491.89	−0.66	0.51	[−1295.46, 645.6192]
OMN	306.8	306.43	1	0.318	[−297.7998, 911.4071]
PAK	493.49	426.25	1.16	0.248	[−347.5393, 1334.529]
PAN	366.84	392.24	0.94	0.351	[−407.0808, 1140.766]
PER	271.73	222.84	1.22	0.224	[−167.9554, 711.4218]
PHL	−104.64	262.64	−0.4	0.691	[−622.8616, 413.5793]
PNG	297.34	316.28	0.94	0.348	[−326.6992, 921.381]
POL	297.22	20.63	14.4	0 ***	[256.5053, 337.9304]
PRT	−41.92	222.84	−0.19	0.851	[−481.6021, 397.7661]
PRY	−60.38	68.79	−0.88	0.381	[−196.1178, 75.3495]
PSE	250.98	256.15	0.98	0.328	[−254.4312, 756.3853]
PYF	300.49	308.45	0.97	0.331	[−308.1146, 909.092]
QAT	207.47	166.47	1.25	0.214	[−120.9961, 535.9267]
ROU	−172.6	299.78	−0.58	0.565	[−764.0896, 418.8857]
RUS	55.24	861.24	0.06	0.949	[−1644.06, 1754.53]
RWA	205.69	207.25	0.99	0.322	[−203.2454, 614.611]
SAU	678.99	400.19	1.7	0.091	[−110.6093, 1468.596]
SDN	678.37	630.08	1.08	0.283	[−564.8316, 1921.575]
SEN	213.10	229.29	0.93	0.354	[−239.313, 665.5046]
SGP	−1055.88	1389.32	−0.76	0.448	[−3797.131, 1685.373]
SLE	236.47	236.93	1	0.32	[−231.0028, 703.9444]
SLV	371.88	399.40	0.93	0.353	[−416.1667, 1159.925]
SMR	211.90	195.89	1.08	0.281	[−174.6041, 598.398]
SOM	300.16	297.33	1.01	0.314	[−286.5072, 886.8234]
SRB	94.44	55.08	1.71	0.088	[−14.23795, 203.1249]
SSD	400.82	407.84	0.98	0.327	[−403.8719, 1205.521]
STP	193.51	198.63	0.97	0.331	[−198.4036, 585.4259]
SUR	243.73	262.98	0.93	0.355	[−275.149, 762.6014]
SVK	−247.02	326.19	−0.76	0.45	[−890.6272, 396.5884]
SVN	19.56	13.68	1.43	0.155	[−7.434995, 46.55195]
SWE	−393.60	866.62	−0.45	0.65	[−2103.527, 1316.32]
SWZ	242.6	251.98	0.96	0.337	[−254.5818, 739.7901]
SYC	132.13	125.70	1.05	0.295	[−115.8824, 380.1409]
SYR	359.9	377.63	0.95	0.342	[−385.2012, 1104.996]
TCD	188.54	157.18	1.2	0.232	[−121.5861, 498.6678]
TGO	238.09	240.84	0.99	0.324	[−237.107, 713.2928]
THA	−888.23	1271.91	−0.7	0.486	[−3397.813, 1621.363]
TJK	244.53	256.35	0.95	0.341	[−261.2624, 750.32]
TKM	241.53	249.09	0.97	0.334	[−249.9408, 732.9929]
TTO	233.84	258.47	0.9	0.367	[−276.1362, 743.8105]
TUN	390.26	415.38	0.94	0.349	[−429.3194, 1209.847]
TUR	151.52	355.53	0.43	0.67	[−549.9788, 853.0178]
TZA	363.15	384.23	0.95	0.346	[−394.9798, 1121.274]
UGA	338.52	352.60	0.96	0.338	[−357.1915, 1034.23]
UKR	−625.16	818.54	−0.76	0.446	[−2240.213, 989.8846]
URY	−251.19	310.16	−0.81	0.419	[−863.1708, 360.7852]
USA	8620.85	3545.36	2.43	0.016 *	[1625.561, 15616.13]
UZB	−500.93	603.00	−0.83	0.407	[−1690.688, 688.8321]
VEN	−229.32	437.64	−0.52	0.601	[−1092.822, 634.1773]
VNM	−357.61	489.28	−0.73	0.466	[−1323.01, 607.7872]
VUT	180.37	171.08	1.05	0.293	[−157.189, 517.9312]
WSM	146.34	140.63	1.04	0.299	[−131.1335, 423.8183]
YEM	343.20	357.33	0.96	0.338	[−361.8306, 1048.234]
ZAF	−490.76	832.22	−0.59	0.556	[−2132.81, 1151.288]
ZMB	194.33	185.64	1.05	0.297	[−171.9579, 560.6236]
ZWE	272.31	291.27	0.93	0.351	[−302.3891, 847.0155]
_cons	−701.87	663.58	−1.06	0.292	[−2011.17, 607.42]

Notes: “***” represents *p* < 0.001, “**” represents *p* < 0.01, and “*” represents *p* < 0.05. The intercept term of each country basically fails the test, which further shows that the individual effect between countries is not significant, and the mixed regression model is, thus, better.

**Table A8:** Results of the RE Model.

Variables	Coef.	Robust Std. Err	Z	*p*	95% Confidence Interval
DTN-NODF	−1.89	1.33	−1.42	0.156	[−4.50, 0.72]
Comp.	46.55	13.81	3.37	0.001 **	[19.49, 73.61]
Intercept Term	−430.93	188.57	−2.29	0.022 *	[−800.53, −61.33]

Notes: “***” represents *p* < 0.001, “**” represents *p* < 0.01, and “*” represents *p* < 0.05. DTN-NODF fails the test, indicating that the fitting effect of random effects is not good.
